# Foreign Correspondence of the Western Journal of Medicine and Surgery

**Published:** 1850-08

**Authors:** 


### WESTERN JOURNAL

OF

### MEDICINE AND SURGERY.

AUGUST, 1850.

---

ART. I.—*Foreign Correspondence of the Western Journal of Medicine and Surgery.*

PARIS, FRANCE, July 6, 1850.

On the 12th of March, 1850, Marie B———, thirty-one years old, and of a bilious temperament, entered the Hotel Dieu. Her father and mother, who died at the respective ages of sixty-three and fifty-nine, were neither of them of delicate constitutions. She has had three sisters, one of them died when forty years of age of a disease of the chest; another a few days subsequently to a fall; whilst the third is still living, the mother of fine healthy children.

Her father died when she was seventeen years old, since which time her condition has been anything but enviable—she has occupied a very small, but generally dry room, high in the building, and exposed to the sun—working from six in the morning until ten or eleven at night, and that week in and week out, with no rest on Sunday. She has considered herself fortunate if she could walk out once or twice a month. Though not stinted in her food her appetite has been extremely small.

When five or six years of age she had the small pox. At the age of eleven the menstrual discharge appeared for the first time, and without pain—ever since it has returned with tolerable regularity, the epoch generally embracing some four or five days and continuing about eight days. It has very often contained clots of blood about the size of a pea, and has always been of a normal color. She has never been the subject of leucorrhea, and has never spit blood—unusual exertion speedily takes away her breath—during the last four or five years this embarrassment has increased, accompanied by palpitations of the heart. During the last three years her digestive powers have become considerably impaired, her strength has diminished, and she has not been able to do as much work as formerly, though occupied nine or ten hours a day, in addition to performing her little domestic duties.

In the month of June, 1848, her courses, accompanied by slight pain in the lumbar and hypogastric regions, continued some two or three days longer than usual, and contained quite a number of coagula of a deep color, and as large as a walnut—but there was no difficulty in urinating, no constipation, no feeling of weight in the fundament. To what to attribute this hemorrhage she cannot tell, but is certain it was not caused by pregnancy, for she is still a virgin—(where is Bernema?) During the ensuing three months the periodical discharge took place as usual and without being accompanied by pain, but in the month of October, some days before its appearance she suffered severe pains which very much diminished the first day of the discharge, only to return with increased violence the second—for five days she was confined to her bed, clotted blood flowing freely the whole time. Since this period she has suffered more or less constantly with pains in the loins, which three days before each menstrual period become very severe, but not sufficiently so to interrupt her daily vocations.

Seven months since the menstrual flux continued for twenty-two days, it was very abundant, mixed with small clots and accompanied by exceedingly severe pains in the lumbar and hypogastric regions and in the groins—her appetite never great, diminished, she became pale and thin, the palpitations increased in frequence and intensity, and during the whole time she was forced to rest in her bed. For two months she was unable to resume her work. For the last three or four months the patient has frequently suffered from sharp lancinating pains on the inside, and sometimes throughout the entire thigh, cramps in the calves, pain in the back, and sleep disturbed by hideous dreams, the digestive function has continued impaired, the appetite has still farther diminished, alternately constipated or laboring under diarrhea, there is often a weighty bearing down sensation in the fundament, a frequent desire to urinate, and occasional nausea. The palpitations have become very frequent, and the breathing very short, but there is an absence of cough, night sweats, or pain between the shoulders.

March 12.—The patient is suffering to an almost incredible degree, eyes bright, face expressive of pain and anxiety, skin of the usual temperature, pulse one hundred and nine, of medium force; respiration rapid. She complains of great pain in the loins and lower portion of the abdomen, but this pain is not much increased by pressure. Abdominal examination does not show any enlargement of the uterus or its dependencies—and asserting her virginity she objects to an examination per vaginum. The menstrual discharge is now present, though not very abundant. The tongue is clean, no thirst, anorexia, and bowels natural. There is a slight souffle in the first beat of the heart.

17.—The pain having commenced at the same hour for the last two or three days, the patient will take six grains of quinine.

28.—No change, the discharge still continuing the patient will take, in a cold injection, forty grains of rhatania. On the 28th the discharge disappeared.

April 18.—Though at no time free from pain the patient complains of its great increase since yesterday evening. It is confined to the lumbar region, principally of the right side, and to the hypogastrium. There are lancinating pains in the thighs, and cramps in the calves. Her courses reappeared this morning.

23d.—The patient is entirely without appetite, the pains still continue, and the discharge very abundant, pale and free from clots is still unarrested. Worn out by pain and anxiety she at last consents to a vaginal examination. Owing to the narrowness of the canal and the patient's repugnance to the examination, the finger was introduced with difficulty, and when about an inch within the vagina, comes in contact with a tumor which at first, owing to the pain and want of size of the parts, it was impossible to circumscribe. By persevering, the finger was carried up behind the tumor as high as the neck of the womb, which was about one and a half inches in diameter. The tumor which was impacted in the orifice of the matrice, filled the whole of the vagina, and seemed even to have dilated it—it was rounded, soft and fluctuating to the touch. On withdrawing the finger it was covered with an extremely fetid and bloody discharge. The patient has fallen away considerably since her entry into the hospital, her face is pale and sharp, and her eyes sunken n their orbits—though she can turn herself readily in her bed, it is more than a month since she has been able to leave it for any purpose. The pain and hemorrhage still continue to increase. Numbness and cramps in the lower extremities, the cutaneous sensibility entirely preserved, sleep imperfect and disturbed, palpitations of the heart greatly increased, no cough, no dyspnea, appetite if possible diminished, absence of thirst, occasional nausea, tongue covered with a white coating, difficulty in going to stool even by aid of injections, passes her water frequently, little at a time, and with difficulty. During the presence of the severe pains to which she is unfortunately too subject, no matter how urgent may be the call or how great the effort she cannot empty either bladder or bowels.

May 4.—The tumor was removed this morning, and was unfortunately lost without having been microscopically examined. It was rounded in form, soft, and fluctuating to the touch, very large, filling the whole vagina so that it was necessary to remove it piece-meal, and attached by a pedicle as large as two fingers, inserted within the mouth of the womb. It was soft, friable, infiltrated, but not bloody, but not having seen the operation, which was performed a day sooner than intended, I cannot be responsible for the description of the piece.

The eight days following the operation passed without anything unusual occurring.

24th.—Fetid and abundant leucorrhea, bowels open every two or three days, disgust for food, and nausea when she forces her appetite, urinates rarely, numbness of the lower limbs, cramps in the calves, and darting pains on the inside of the thighs, prostration.

26th—The menstrual discharge appeared to-day, eight days sooner than the last time, discharge slight.

31st.—Discharge ceased to-day, pain in the hypogastrium, where by pressure a tumor may be perceived. A vaginal examination shows the neck of the womb to be in its right direction and of its proper consistence. No trace of the tumor remaining.

June 2.—Day by day she is gradually sinking. Numbness in the lower extremities, painful cramps in the calves, which without continuing long at a time return frequently. Neither trembling, convulsive movements or edema. No pain in the tracks of the large nerves. Pressure does not increase the suffering. Diarrhea; pulse eighty-eight; respiration twenty-four.

9th.—Complains of itching of the hands and forearms, numbness of the extremities continues, slight difficulty in moving the head upon the shoulders without any rigidity of the muscles of the neck, head free from pain, vision distinct, hearing unimpaired, and deglutition perfect, entire freedom from pain in the hypogastrium, though upon pressing in that region an enlargement of some of the contained organs is readily perceived—it is immediately above the pubis and extends right and left.

24th.—Increased prostration, sensibility entirely unimpaired; patient rapidly sinking.

25th.—Dead.

Autopsy twenty-four hours after death:

There is neither adhesions or effusion in the pleural cavity. The lungs are shrunken, and at the summit of each are a few crude tubercles. The heart presents nothing abnormal. The peritoneal cavity is perfectly healthy above its pelvic portion. The central part of the transverse arch of the colon is drawn down behind the pubis. The peritoneum contained in the pelvis is all more or less inflamed, and all of the organs covered by it are more or less adherent to each other. The adhesions however were soft and readily broken up, more an agglutination than what we understand by *adhesion*. The bladder is healthy. The ovaries are connected by adhesions to the small intestines, their peritoneal covering much thickened, and their substance perfectly riddled by numerous collections of a greenish-yellow pus. The fallopian tubes are thickened. The uterus about double its normal size, is almost at right angles to its natural position, its long diameter extending between the pubis and the upper portion of the sacrum, the fundus immediately behind the symphysis. The lining membrane of the organ is of a brownish-red color, and the point of insertion of the pedicle of the removed polypus just within the os tincea, is of a dark brown—immediately by the side of this is a small rounded tumor about the size of a small cherry, and entirely within the cavity of the uterus. It is firm without being hard, of a pale pinkish color, and pediculated—submitted to the microscope, no malignant structure found, it is entirely composed of fibro-plastic elements.

Spleen, liver, and kidneys all healthy.

Brain perfectly normal. The spinal marrow examined throughout its whole length was found softened in the lumbar region, reduced to the consistence of curds—almost as soft as the thickest cream, and of an uniformly dead white color. In its dorsal and cervical portions it was as usual.

